# Monograph of the Class Myriapoda, Order Chilopoda; with Observations upon the General Arrangement of the Articulata

**Published:** 1845-10

**Authors:** 


### Art. XIX.

1. *Monograph of the Class Myriapoda, Order Chilopoda; with Observations upon the General Arrangement of the Articulata.* By George Newport, Esq. Fellow of the Royal College of Surgeons, President of the Entomological Society, &c. 4to, pp. 38. With a Plate. (*From the Transactions of the Linnæan Society,* vol. XIX, 1844-45.)

2. *On the Structure, Relations, and Development of the Nervous and Circulatory Systems, and on the existence of a complete Circulation of Blood in Vessels, in Myriapoda, and Macrourous Arachnida. First Series.* By George Newport, Esq. f.r.c.s. &c. &c. 4to, pp. 60. With Five Plates. (*From the Philosophical Transactions,* 1843.)

3. *On the Reproduction of Lost Parts in Myriapoda and Insecta.* By George Newport, Esq. f.r.c.s. &c. &c. 4to, pp. 12. With a Plate. (*From the Philosophical Transactions,* 1844.)

We trust that few of our readers are unacquainted with the name and general merits of Mr. Newport, who has been devoting a large amount of time and labour, during a long series of years, to the investigation of the anatomy and physiology of articulated animals of various classes; and this in the midst of difficulties of various kinds, which would speedily have baffled and dispirited a less earnest and persevering inquirer. But it is not earnestness and perseverance alone, that can make a *discoverer;* even in a branch of science on which so little was known, and so much lay open to investigation, as that to which Mr Newport has devoted himself. Great manipulative skill and sharpness of vision are required, for the performance of dissections of such excessive minuteness; and above all a sagacious, penetrating, retentive, intellect, is necessary for the direction of the eyes, and the guidance of the hand; with reflective and generalizing powers to compare, arrange, and combine the facts ascertained; and a fearless love of truth, that shall lead to the ready abandonment of erroneous conceptions, however cherished, when once they are found to be inconsistent with facts. Such qualities, and many more of similar value, Mr. Newport possesses; and we cannot but regret that the limited patronage, which it is the custom of the Government of this country to afford to scientific men, at least when their discoveries have not an immediate *practical* value, has not yet been extended to him. In now presenting our readers with a somewhat detailed account of Mr. Newport's most recent contributions to the anatomy and physiology of a class of animals to which little attention has been hitherto directed, we are influenced mainly by the conviction that the discoveries have a strong claim upon the attention of our readers, from the importance of the principles they contribute to establish in *general* physiology. That portion of his labours relating to the Nervous System, formerly noticed by us (vol. XVII, p. 181, et seq.), affords a pregnant instance of this most important relation of Mr. Newport's investigations to the every-day pursuits of practical men.

We shall commence with a few observations on the general structure, and history of the development, of Articulated animals in general; by which the subsequent details may be rendered clearer to those who have no previous acquaintance with the subject. The general character of the group is derived from the inclosure of the body in a firm envelope, divided into segments by transverse lines, as is best seen in the centipede. These segments are extremely numerous in the lower forms of the group, such as the tape-worm, and some of the marine annelida; and there seems to be even a considerable indefiniteness about their number; they are comparatively few, however, in the higher classes, and their number is always the same in each species. The longitudinal repetition of similar parts is not confined to the external or tegumentary system; for it is more or less characteristic of all the internal organs, especially in the lowest vermiform animals. Thus in each joint of the tape-worm we have not merely the nutritive, but the generative apparatus repeated on the same plan; and among many of the marine annelids, we find that the external appendages present an exact uniformity throughout, that the nervous system exhibits a corresponding multiplication of distinct but similar centres, and that the circulating and respiratory systems show indications of the same segmental independence. Now, in the development of these animals, we have a most extraordinary proof of this independence. It has long been known that in the tape-worm, the head and a few segments might continue to live, after being detached from all the rest of the body; and that new segments would then be continually added, by a sort of *budding* process, until the number might reach several hundreds; but recent observations, especially those of M. Milne-Edwards, (Annales des Sciences Naturelles, Mars 1845,) have shown that this is the *normal* method of development of many marine worms, which come out of the egg with nothing but a sort of head, behind which segment after segment is developed, until the full length is attained; the animal, at an early stage of this process, bearing so strong an analogy to certain of the wheel-animalcules (rotifera,) as to leave no longer any doubt as to the zoological affinities of the latter class. In these instances, then, we have striking examples of the simplest form of development, distinguished by Mr. Newport as that of *growth,* which consists in the mere extension of the original structure by the progressive enlargement of the old parts, or the addition of new ones similar to them. Now, in the classes which are nearest to the opposite extremity of the series, we find a very different series of phenomena. Instead of the *segmental equality and independence,* which are such striking features in the conformation of the vermiform tribes, we meet with a continual *inequality,* some being highly developed at the expense of others, and some possessing appendages of which others are destitute; and we also find that many segments undergo a more or less complete fusion or *aggregation,* so as to form a composite structure, in which their individuality and independence are almost completely lost. Yet by the study of the history of development in these classes, it may be seen that the segmental independence is at first as complete (or nearly so,) as in the lower classes; but that the process of simple *growth* soon ceases, ending with the production of a comparatively small number of segments, and being then superseded by the process of *aggregation.* "This latter mode of development is carried to a greater extent in insects than in any other invertebrata; and changes the animal, from a simple, elongated, worm-like larva, furnished with many pairs of organs of locomotion, and composed of segments almost uniform in size and appearance, to an individual of a totally different character, with its body divided into three regions, differing in size and appearance, and separated from each other, with the number of its organs of locomotion reduced, and those that remain greatly altered and enlarged."

The class of Myriapoda holds an intermediate rank, both in structure and development, between the two extreme forms which we have noticed. It consists of two groups: the higher termed the *Chilopoda,* and consisting of animals allied to the centipede in external form; and the lower, or *Chilognatha,* containing the iulus or galley-worm, and its allies. In the former, which is carnivorous, the body is mostly flattened and very flexible from side to side, whilst the legs are well developed, and serve as the instruments of locomotion. The segments and limbs are much less numerous than in the latter group; in which the body is usually almost cylindrical and flexible in any direction, the number of segments being very considerable; and the legs, though very numerous, being weak and thread-like, and being destined rather to assist in progression than solely to accomplish it. Altogether it is evident that the iulidæ are much more allied to the inferior vermiform tribes; and the scolopendridæ to the higher groups of articulata. This appears also from the history of their development, which Mr. Newport has carefully studied. (Philosophical Transactions, 1841.) The *iulidæ* do not come forth from the egg in a condition quite as low as the marine annelids; but the number of segments is out of all proportion to that which the animal is afterwards to possess, being only *eight,* and being subsequently increased even to *ten times* that number, so that the process of *growth* is evidently here predominant. On the other hand, in the *scolopendridæ,* whilst the production of new segments never goes on to anything like this extent, a considerable degree of *aggregation* takes place. A certain amount of this occurs, however, in *both* orders of myriapods. The head is composed of several segments, (*eight,* according to Mr. Newport;) "either consolidated together like the head in true insects, as in the vegetable-feeding chilognatha; or separated and moveable on each other to adapt them to the carnivorous habits of the rapacious chilopoda. In like manner, each moveable division of the body is in reality composed of two distinct segments, originally separate, but anchylosed together at an early period of their formation. Each of these sub-segments ought therefore to possess its separate ganglion and pair of legs; and this is usually the case. The degree in which this coalescence affects the internal organs, varies greatly in the several families of the class; being much greater, as we shall hereafter see, in the scolopendridæ than in the iulidæ." In the polydesmidæ (which form part of the latter group,) the ganglia remain distinct throughout life in the posterior segments, but coalesce in the anterior, more especially in those nearest the head; so as to foreshadow, as it were, the much greater change which takes place in the thoracic region of insects.

The *Myriapoda* were first raised to the rank of a distinct class by our own countryman, Dr. Leach; and this determination has been generally adopted by recent naturalists, with the exception of M. Brandt, who still attaches the group to the class of insects. But the place to be assigned to it has been a more questionable matter; and many have been the differences in the views on this subject adopted by the several zoologists, who have paid special attention to the group, or who have been engaged in the construction of general systems. Although Linnæus did not recognize them as a distinct class, he seems to have had a clear notion of their true position in the scale; for he placed them at the end of the apterous insects, immediately before the true vermes,—an arrangement which, as Mr. Newport justly remarks, is in full accordance with the facts now ascertained respecting their metamorphoses and mode of growth, which indicate their close affinity to the latter class. Many subsequent classifiers, however, struck with the resemblance between certain myriapoda and crustacea, on the one hand, and certain myriapoda and arachnida on the other, have placed them *between* these two classes, which are certainly much more nearly related to each other than to the myriapods. Of late, the tendency has been to place them in close alliance with the wingless insects; on which Mr. Newport makes the following remarks:

"After an attentive examination of the myriapoda, as compared with other articulata, I have been unable entirely to adopt the views of any one of the distinguished naturalists above noticed, either in regard to the situation which they ought to occupy in the arrangement of the invertebrata, or to the affinities by which they are connected with the other classes. They certainly have many close relations to the larva state of true insects in the elongated form of the body, in their mode of respiration, in the structure of the organs of circulation and nutrition, and also in the arrangement of their nervous system; but they differ from them entirely in their mode of growth and development.

"The *myriapoda* acquire a periodical addition of segments and legs, with their separate ganglia, nerves, and other structures. This addition of new parts, at each change of tegument, takes place in all the myriapoda up to a certain period of their growth, which period varies in the different genera. But this addition of parts never occurs in insects, even in the lowest forms of the class, or even in their earliest stages, after leaving the ovum. Every entomologist is aware, that when an insect bursts from the egg, it is furnished with the whole number of segments and legs it is ever to possess; and in no instance does the number of segments exceed fifteen. The usual number, *thirteen*, as naturalists are well aware, is very rarely exceeded; although in some of the hymenoptera, Mr. Westwood and myself have observed fifteen. During the changes of the insect, this number is gradually reduced, by the aggregation and anchylosis of some of the segments to form particular divisions and regions of the body, in the construction of which some of the segments become enlarged, and others are atrophied or almost obliterated. In the *myriapoda*, on the contrary, the young animal invariably comes from the ovum with its smallest number of segments, and in most of the genera this seldom exceeds *nine;* although before the myriapod has arrived at its full growth, it acquires in some species nearly eight times the original number; a definite number of new segments being constantly in the course of formation between the antepenultimate and penultimate segments of the body. This is the great characteristic of the class, which distinguishes the *myriapoda* from *insecta, arachnida*, and *crustacea*, and approximates them to the annelida, in which a similar addition of parts takes place. The myriapoda are also distinguished from insects by a permanent anatomical character, the number of segments and legs in the adult animal. These are never fewer than *twelve* segments and *eleven* pairs of legs in any genus of the myriapoda, In some genera, the latter even amount to *one hundred and sixty;* while no insect, even in the larva state, has more than *eight* pairs, five of which are rudimentary, and disappear as soon as the four anterior segments have acquired their full growth, and the insect undergoes its metamorphosis, when its legs are reduced to *three* pairs, and the insect passes into a higher state of development. These are the considerations which have led me, with Leach, Latreille, and others, in opposition to the high authority of M. Brandt, to separate the myriapoda from the true insects, and to place them as a class immediately before the annelida." (Linn. Trans. p. 269.)

The question raised by Mr. Newport as to the order in which the classes of the sub-kingdom Articulata should be arranged, is one of much physiological as well as zoological interest. He considers that the insects ought to stand at the head of the series, that the arachnida should follow, then the crustacea, next the myriapoda, and next the lower vermiform classes. The question is by no means fully discussed by Mr. Newport, however; and we shall pass it by on the present occasion, with the simple expression of our opinion, that it is no more possible to represent the classes of the sub-kingdom articulata, than it it is to arrange those of the whole animal kingdom, in any linear series. We fully agree with Mr. Newport in regarding insects as the *types* of the articulated sub-kingdom, that as presenting its peculiar characters in their fullest development. But the nearest approaches to the vertebrate division are not to be found in that class, but rather among the arachnida and crustacea; the latter of which groups seems to have been directly connected with fishes, by species long since extinct.

We shall now follow Mr. Newport through some of the details of his inquiries. The following general facts should be kept in mind, in all comparisons of vertebrated and articulated animals:

"The nervous cord in the articulata is extended along the ventral surface of the body, and it is that portion in each segment which is first completed; while in the vertebrata it is extended along the dorsal surface, which in like manner first acquires its definite form. The dorsal surface of the articulata is occupied by the vascular system, and, like the abdominal surface, at which the nutrient vessels of the body enter, in the vertebrata, is the last portion of the external surface of the body that is completed, as may be readily seen in the development of the animal in the ovum. Consequently the dorsal portion of the tegument in the myriapoda, and other articulata, is less early completed than the ventral, although often developed to a much greater extent." (Linn. Trans. p. 281.)

The following statements regarding the varying modes of development in the different subdivisions of the class, are very interesting:

"In the *chilognatha*, the normal segments produced at each change of tegument remain perfectly distinct throughout life, and only acquire their full size by the first mode of development,—simple growth. But even in the lowest forms of chilognatha, in which this first mode is chiefly predominant, the second mode also, the coalescence or anchylosis of two approximated normal segments, takes place almost at the period of their formation. But the original distinctness of the two continues marked throughout the whole life of the animal, so that each moveable segment of the body is formed of two distinct normal *subsegments*. Each of these subsegments retains its legs, both pairs being equally developed. This is the condition of the body in the lowest or iuliform chilognatha.

"In the lower forms of the *chilopoda*, the *geophilidæ*, there is a progressive change in the mode of development. This takes place in the ovum. The two subsegments of which each moveable division of the body of the perfect animal is composed, and which subsegments are at first equally developed, not only become anchylosed together before the embryo bursts from the fœtal coverings, but the posterior of the two exhibits a marked superiority of size. This difference continues to increase at each change of tegument, after the animal has left the ovum, until each anterior subsegment has scarcely more than one half the extent of the posterior. This difference is greatest on the ventral surface, where the sternal plate of the posterior subsegment covers nearly the whole. Coincident with the beginning of this change and union of each pair of subsegments in the ovum, only one pair of legs is developed to each compound segment, and these have their origin in the posterior of the two subsegments. Notwithstanding this difference in the extent of their development, the rudimentary portions of the anterior segment still exist in the form of minute, partially-detached plates, at the front of the posterior segment, the dorsal arc being represented by a very short transverse portion.

"In the higher genera of *chilopoda*, as in *scolopendra*, the number of compound moveable segments to the body is greatly reduced, and a further union of the two subsegments has taken place." (Linn. Trans. p. 285.)

The upper surface of each moveable segment in this genus is covered by a single plate, on the anterior part of which is only a slight indication of the original existence of the first subsegment, in the form of a narrow elevated transverse band. The remains of this subsegment are more distinct on the ventral surface; but not even the rudiments of appendages are to be found. Now as this is a clear case of the *unequal development* of the two segments, we think it ought not to be ranked in the same category with *coalescence* or *aggregation;* and should suggest to Mr. Newport whether the modes of development distinguished by him, should not be three rather than two.

"It is in this way, by changes that take place in the relative development of the rudimentary segments of the embryo in the ovum, that each animal is originally formed on a comparatively higher or lower type, according to the greater or less extent of change which the embryo undergoes in its earliest stages. The form impressed on the future animal, when these changes in the ovum begin to be arrested, is usually that by which its further development is to be regulated; and which it may retain either as a permanent condition, or only as a form that requires to be further matured in post-embryonic life before it is fitted to take that which it is ultimately to assume. It is in this way that the coalescing segments of *geophilus* become further united in *scolopendra*, and are completely lost in single structures in *lithobius* and *cermatia;* in each instance the union of the rudimentary segments taking place in the ovum, and the type of formation then impressed on the animal being afterwards uniformly repeated at each change of tegument and production of new segments.

"The mode in which development takes place, by a union of similar parts, is always *centripetal.* When any portion of the body has acquired its fullest extent by the *first* mode, that of simple growth or enlargement, it acquires a tendency to coalesce or become united with similar adjoining structures, either by simple anchylosis of the two, or by a greater or less extent of direct union or coalescence; and the two parts which thus become joined, tend to one common centre.

"What takes place in regard to individual structures, takes place also in the whole body; as is shown in the transformations of insects. While some segments of the body of an insect become more or less completely approximated in sections, and divide the body into regions, the whole exhibit the same tendency to approach each other; the head is applied more closely to the thorax, and the thorax is approached by the abdomen.

"These, then, are the principles, on which the body of an articulated animal is developed, and acquires its proper form and dimensions; and which are carried to their greatest extent in hexapod insects. They seem to prepare the way for a higher type of development, at a much earlier period of the ovum, of the vertebrata, and to lead to the permanent division of the body, in the more perfect animals, into important regions,—the head, thorax, and abdomen." (Linn. Trans. p. 287.)

Mr. Newport then proceeds to inquire into the structure and development of the head of Myriapoda; from which he believes (and we fully accord with him) that the most certain indications may be drawn in regard to the normal number of segments entering into the composition of this part in articulated animals. Many attempts have been made to ascertain this fact, by examining the head in hexapod insects; but in consequence of its higher type of development and more compact form in that class, the results arrived at by the several naturalists who have engaged in the inquiry, are by no means uniform. The different conclusions seem to have arisen from the fact, that the number of segments is in reality more considerable than has been usually supposed; and that, in certain species which have been examined, every trace of some of the segments has disappeared; whilst the very same parts may be enormously developed, and others atrophied, in a different genus. On this inequality depends the form of the organ. Thus Burmeister recognizes but *two* segments; Carus and Audouin *three;* Macleay and Newman *four;* and Strauss-Durckheim as many as *seven.* But Mr. Newport now adduces strong evidence, that even this last number is insufficient; and that we must reckon *eight* as the real amount. This result is attained, by tracing the changes of the myriapod from the ovum, and by comparing the adult forms of the different genera; and the same mode of investigation fully confirms the original views of Savigny, that the parts of the mouth are the analogues of the organs of locomotion, and acquire their various forms in consequence of the different extent to which their individual parts are developed. Thus, in the Chilopoda, the head is formed of two moveable distinct portions, well seen in the Scolopendra; the anterior of these is denominated the *cephalic,* and the posterior the *basilar* portion. Each of these is originally composed of four subsegments; as may be most distinctly traced in the inferior genera of that order, in some of which the parts composing the basilar division never completely unite, but those forming the cephalic division coalesce at the time of the emersion from the egg. Each subsegment, like the segments of the body, normally bears a pair of appendages of some kind. That the *first* bears the antennæ, which are largely developed in geophilus, whilst the segment itself is atrophied. The *second* does not give origin to moveable appendages, but contains the eyes; it is more developed than the first, and is almost entirely occupied by the great centre of the animal functions and instincts,—the brain. The *third* is developed to a greater extent than the second; and gives origin to appendages which are the first moveable parts of the organs of nutrition, the internal maxillæ. The *fourth* subsegment is equal in length to the whole of the three anterior segments; and bears as its appendages certain large, three-jointed, palpiform organs, which seem to represent the external or maxillary palpi of insects. Shortly after the animal has left the ovum, the cephalic segments are nearly all of the same size; but the first soon shows a retardation, and the fourth an acceleration, of growth. The *fifth* subsegment, which is the first that enters into the basilar portion, bears the labial palpi; and the *sixth* bears the large forcipated mandibles. "The mandible, at the bursting of the egg, is only a simple tubercle to the sixth segment of the head, precisely similar in every respect to form and size, to the tubercles of other segments of the body, which afterwards become legs or organs of locomotion, &c. But during the short space of time that elapses while the embryo is escaping from the egg, and before it has rid itself of the fœtal membrane, this little tubercle is enlarged to twice the size of the others, and continues to increase rapidly; at the same time undergoing a change in the relative development of its parts, which so modifies its whole form, as to adapt it for the function of prehension and manducation, instead of locomotion." The central portions of the fourth, fifth, and sixth segments enter into the composition of the mouth. The *seventh* and *eighth* subsegments generally unite at an early period, and sometimes coalesce with the anterior segments; their appendages are sometimes developed as true legs, and are sometimes atrophied, still however showing traces of their regular existence; but in the lithobius these are altogether wanting, the whole basilar region being reduced on the dorsal surface to a narrow ring, and the cephalic region being enormously developed.

We have entered so far into the details of this curious investigation, as an illustration of the necessity of prosecuting any such inquiry regarding the elements of the cranium of vertebrata, on the method which Mr. Newport has so successfully adopted and carried out;—namely, by selecting the group in which the parts are most distinct, and then comparing the several variations which the adult forms present, with the more uniform type manifested in the embryo. We anticipate most interesting results from Mr. Newport's application of the same method of inquiry to the composition of the head in insects. It would be quite beyond our province to enter into those details of the external anatomy and zoological arrangement of the myriapoda, which make up the remaining part of this paper: we may, however, state, in passing, that the paper is a complete monograph of the class, and that, besides establishing several new families and genera, Mr. Newport has here minutely described about 140 new species, and given many important observations on their natural history and habits.

The next on our list relates to the anatomy and physiology of the Nervous and Circulating Systems in this class, and also in the Macrourous Arachnida. The portion of this which relates to the nervous system of the myriapods, has passed under our review on a former occasion, (vol. XVII, p. 181 et seq.) so far, at least, as regards its bearing upon the doctrines of reflex action. We have now, therefore, only to notice the facts relating to the *development* of the nervous system; which, for the reasons already mentioned, can be studied particularly well in this group.

The account of the posterior portion of the nervous system in *polydesmus maculatus* affords the most information on this subject. In this animal, the number of segments is twenty-two, including the head and anal segments; and the number of distinct ganglia in the cord is thirty-four, each supplying one pair of organs of locomotion. The anterior portion of the cord presents some modifications, partly connected with the generative apparatus; but posteriorly to the fourth segment in the female, and the seventh in the male, the cord is extended backwards, nearly in a uniform manner throughout the remaining segments, as far as the thirty-second ganglion, when it becomes less uniform. In the first part of its course, it forms two ganglia in each segment; the interval between these being about half that which exists between the last ganglion of one segment and the first of the preceding. Each ganglion sends a pair of nerves to the legs; but the trunks which supply the muscles and sides of each segment are given off from the cord, midway between the two ganglia. In proceeding backwards along the cord, however, the distance between the ganglia of each segment is gradually lessened, and at last they almost unite; and the nerves which came off from the cord between the ganglia are found to arise nearer and nearer the ganglion next behind, until they cease to come from the cord, but are derived directly from the ganglia. But although the ganglia are thus closely collected together, this is not the result of *aggregation* in this part of the body, but is consequent upon the non-completion of those changes which take place in the formation of new ganglionic centres and nerves in this part of the cord, and which are carried to a much greater extent in the iulidæ. These formations always take place between the penultimate and antepenultimate segments; and it is in that part of the cord, therefore, that the new ganglia are produced to those segments. When comparing the ganglia, cord, and nerves, therefore, in the segments of most recent formation, with those of the segment next anterior, and so progressively forwards, we are in reality studying the progressive stages of development of these parts. From such a comparison, Mr. Newport arrives at the following conclusions:

"The cord is elongated *in* the ganglia, by extension or growth longitudinally; and those nerves which are given to the sides of the segments and to the respiratory structures, and which originally are formed in the ganglia or in immediate connexion with them, are gradually separated from them, and are afterwards attached only to the interspaces of the cord, so that they are removed to a greater distance from the ganglia, in proportion to the earlier development and more complete state of the segment to which they belong. This elongation of the cord commences in the posterior ganglion, at the front of which, apparently by separation of part of its own structure, the new ganglion of each last-formed rudimentary segment is always produced. Hence the ganglia must be regarded as performing a most important office in the nervous system, that of being centres of growth and nutrition to the cord and nerves. The structure of the ganglia confirms these conclusions, and shows that not only are these parts centres, in which the reflected motions of the limbs are effected, but that they are even of more importance, being those in which the structures themselves are nourished. The vessels distributed over the ganglia penetrate into their substance, and are more abundantly supplied to them than to any other parts of the nervous system." (Phil. Trans. 1843, p. 256.)

There is another point of great importance in relation to this subject, which we must here notice more particularly. In our former notice of this portion of Mr. Newport's paper, having been exclusively concerned with his discoveries on the structure of the ventral cord and his experiments on reflex action, we overlooked his brief but very interesting remarks on the structure and development of the cephalic ganglia, which are commonly spoken of as the brain. To the brain of vertebrata as a whole, however, they cannot be justly regarded as corresponding; their real analogy, first pointed out by Mr. Newport in 1838,* being to the corpora quadrigemina. In the head of the myriapod, the *first* pair of ganglia is of small size, and its branches are distributed to the antennæ; and it is the second pair, immediately behind them, which is obviously the principal centre of the sensori-motor portion of the nervous system. These give off

* Observations on the Anatomy, Habits, and Economy of *Athalia Centifoliæ*.

nerves to the eyes; but are more highly developed than the antennal ganglia, even when the organs of vision are entirely wanting, as in the polydesmidæ. Their relative size increases, as we ascend to the higher classes; and a remarkable increase presents itself during the metamorphoses of insects. The following statement in regard to the development of these ganglia, appended by Mr. Newport in a note, is of great interest.

"Since this paper was delivered to the Royal Society, I have found that, in the embryo of geophilus longicornis, at the moment of bursting its shell, the brain is composed of *four* double ganglia, the centres of a corresponding number of segments, which are then becoming aggregated together to form the single moveable portion of the head in the perfect animal; so that the brain of the myriapod, and probably also of all the higher articulata, is, in reality, composed of at least four pairs of ganglia." (Phil. Trans. 1843, p. 245.)

This fact is one of much importance, not merely from its bearing upon the composition of the cephalic ganglia, in those articulata in which all trace of the original distinctness of their component parts is lost, but also as indicating the probability of a similar process of coalescence in the early development of the brain of vertebrata. It is well known that such a mode of accounting for the arrangement of the cerebral nerves, and of the bones of the cranium, has been proposed by various philosophical anatomists; but it has been treated by those incapable of comprehending it aright, as a visionary piece of transcendentalism. The fact being thus established by Mr. Newport, however, in regard to a group of animals in which it had not been at all suspected, the probability obviously becomes much stronger in the vertebrata. The first ganglion of the ventral cord, which is situated upon the point of junction of the two crura that pass downwards around the œsophagus, obviously corresponds in function, not to the cerebellum, but to the medulla oblongata of vertebrated animals; and this has been found by Mr. Newport to be formed by the coalescence of the first four subœsophageal ganglia, belonging to the four segments that constitute the posterior portion of the head.

We now pass on to Mr. Newport's account of the Circulating System in the Myriapoda; a department of comparative anatomy which has hitherto been very ill understood, but on which he has thrown all the light that skill and industry could afford; so as, in fact, to have most completely elucidated it. The existence of a motion of the fluids of the articulata has long been known to the microscopic observer; but notwithstanding this, and the evidence of a distinct pulsatory action of the great dorsal vessel, as seen through the tegument in the transparent larvæ of insects, and notwithstanding, too, that the circulating apparatus of the higher crustacea had been proved to be most complete, the existence of a true circulation in insects and other air-breathing articulata was doubted, until the fact was demonstrated by Carus and confirmed by Wagner and others. Yet the means by which this circulation is carried on, whether in vessels with distinct parietes, or in sinuses bounded by the other structures of the body, is still a matter of inquiry; and the existence of vessels in insects has recently been denied by no less an authority than Leon Dufour. The most important contribution to our knowledge on the subject, up to a recent period, was that of Strauss-Durckheim; who, in 1825, discovered the existence of distinct chambers and valves, with lateral orifices, in the dorsal vessel of insects; but he was unable to discover any vessels connected with, or proceeding from that trunk. In the myriapoda he found that the anterior portion of this structure in the scolopendra divided into three branches, which are distributed to the head; and that the middle one of these gave off other branches, the course of which he was unable to trace. Previously to this, in 1812, Treviranus had discovered some of the peripheral vessels in the arachnida; and Müller, in 1824, had traced a vascular connexion between the dorsal vessel of insects and the ovaries. Various isolated facts were subsequently contributed, in regard to insects, by Mr. Newport; and in regard to the scolopendra, by Mr. Lord, who was first to demonstrate the vascular character of what is now termed by Mr. Newport the supra-spinal vessel, which had been previously described by Treviranus and Mr. Newport himself, as a part of the nervous system; and by Müller as a ligament. Comparing insect and human anatomy, we might say that the amount of knowledge previously attained related solely to the heart and aorta, and a few isolated trunks in other parts; and that Mr. Newport's merit, in the researches of which we are now going to give an abstract, is as if he had traced out the whole of the arterial system, proceeding from the aorta, a great part of the venous, and (in the scorpion) the whole pulmonary system in addition; besides determining the course of the circulation through the complicated series of vessels he has discovered.

We presume our readers to be aware, that the *dorsal vessel* consists of a longitudinal series of chambers, connected by orifices; each chamber, in fact, representing the heart of its own segment. At each constriction of the heart in the iulidæ, between two chambers, there are two transverse lateral orifices, as in insects, through which the blood enters the organ; these are regarded by Mr. Newport as the orifices of delicate veins, although he is not quite certain whether the veins do not rather deserve the term of sinuses. The heart is inclosed in a delicate membrane, which excludes it from the surrounding structures; this covering ought certainly to be regarded as a pericardium, and not as an auricle, which it was supposed to be by Straus. In all the iulidæ, the heart gives off, in each moveable segment of the body, two pairs of branches, which pass to the sides of the body and to the viscera; these vessels, which have not been hitherto described, are regarded by Mr. Newport as *systemic arteries*. The anterior chamber of the compound heart descends upon the œsophagus, near its termination in the stomach; and from it proceed three pairs of arterial trunks, which pass down on either side of the œsophagus, and unite beneath it, thus forming three vascular collars around this part of the alimentary canal, very similar to those which are more multiplied in some of the annelida. The distribution of the vessels proceeding from these vascular collars is very similar to that which we see in the Batrachian reptiles during their fish-like condition; for whilst some of them pass forwards to supply the head, the main trunks of the first pair unite below the œsophagus to form the great median vessel of the abdomen, which, being situated between the nervous cord and the viscera, obviously corresponds with the aorta of vertebrata. Hence Mr. Newport very appropriately designates this pair as the *aortic arches*.

"In this general structure of the circulatory organs in these vermiform articulata, we perceive a shadowing-out of the great circulatory organs of the higher animals. The ventricular heart, with its aortic arches and great descending aorta, is rudely sketched in this many-chambered great dorsal vessel or heart of the myriapod, with its lateral arches uniting below the œsophagus to form the great channel for the blood to the organs of locomotion and sides of the body—a structure of which the type of formation is continued uninterruptedly, but gradually increasing in complicacy and importance as we ascend through this and the other classes of the articulata, to the lower forms of vertebrated animals.

"In the observations already detailed on the nervous system of these animals, I have shown that each moveable segment of the body is double, and is formed originally of two segments, which are anchylosed together from a very early period of growth; and that, as the segments in the anterior part of the body become more and more nearly approximated, the gangliated portions of the nervous cord in those segments also become closely united. Now what occurs in this respect in the nervous system, takes place also in the vascular. Each double segment of the body in the iulidæ contains, at an early period of growth, two distinct chambers of the heart, each giving off its pair of arterial vessels, and furnished also with its two pairs of lateral muscles. I have found these chambers distinct, and still separated, in *iulus terrestris,* so late in life as that which I shall hereafter have occasion to describe as the *ninth period of development,* when the individual possesses forty-four moveable segments. After that period the *two* chambers in each double segment unite and form but *one* chamber; while the reduplication of the muscular tunics, which form the boundaries of each double chamber, and in which are situated the auricular openings, becomes more complete. Each chamber of the heart, in the adult iulidæ, has therefore two pairs of systemic arteries, and four pairs of lateral muscles. The union of the two chambers seems to be occasioned by the growth and changes induced in the external coverings of the body, at the period when the animal undergoes its semi-metamorphosis, or change of tegument." (Phil. Trans. 1843, p. 277.)

In the *chilopoda,* which form the higher division of the class, the *contractile* tissue of the chambers of the compound heart, as also the great abdominal trunk formed by the union of the aortic arches, are much better developed than in the Chilognatha; and we shall therefore introduce Mr. Newport's descriptions of these parts in the scolopendra or centipede.

"The heart is composed of two distinct contractile tunics, an external and an internal one, each covered by its proper serous membrane. The *external tunic* is covered by the membrane that forms the inner layer of the pericardium. It is a very thick muscular structure, the fibres of which are loosely interwoven with each other. It completely incloses the second or inner tunic—the proper ventricular structure—from which it may be separated without difficulty; and it also forms the external covering of the systemic arteries at their origin, and may be traced along them to some distance from each chamber. The action of its fibres seems to be chiefly in the longitudinal direction, and thus to assist mainly in shortening of the vessel. The *inner tunic* is formed of two sets of muscular fibres. The inner one of these, covered by a delicate membrane, lining the ventricle, consists of longitudinal fibres, which are most perfectly developed on its upper and under surfaces, and which are extended throughout its entire length, from the posterior segment to the head. The other set, which is external to this, is formed of numerous short, broad, transverse muscular bands, very much resembling in appearance the cartilaginous rings of the trachea in vertebrated animals. These transverse muscular bands are thicker and stronger than the longitudinal fibres, and form the sides of this tunic. They do not completely encircle the longitudinal ones, but pass only half way round, on each side, having a space between those of the two sides, both on the upper and under surface. This space is occupied by the principal longitudinal fibres, to the sides of which the extremities of these transverse bands are approximated. They are not, however, all arranged in one parallel longitudinal series, but are placed alternately nearer to, or more distant from, the median line." (Phil. Trans. 1843, p. 280.)

This curious arrangement of the transverse fibres is stated by Mr. Newport to exist in the systemic arteries given off from each chamber of the heart, as well as in the parietes of the heart itself; and he suggests it as a curious point for inquiry, whether it is common also to the arterial trunks in vertebrated animals. It seems difficult to assign a use for it; unless it be to favour that successive peristaltic action, which is here required, rather than the simultaneous contraction, to which a series of complete rings might be expected to be subservient. The auricular orifices formerly noticed are here provided with distinct valves. From the posterior-lateral margin of each orifice, a series of oblique fibres passes diagonally forwards, in the interior of the chamber; until, meeting in the middle line, they form a double valve, with its apex directed forwards, very similar in appearance to the tricuspid valve in the heart of mammalia. This valve is extended forwards from the upper surface of each chamber, a little beyond the outlets of the systemic arteries, and prevents the return of the blood from the anterior part of the chamber.

The great ventral artery,—which lies upon the nervous cord, and is hence termed *supra-spinal* by Mr. Newport (a term that appears to us objectionable, as tending to obscure the analogies of this vessel in the vertebrata,)—originates, as in the iulidæ, from the reunion of the first or principal pair of the arches forming the vascular collar; but it receives branches, also, from the secondary arches. It extends, along the median line, as far as the terminal ganglion of the last segment.

"At its commencement, it is nearly equal in size to the great nervous cords along which it is extended; becoming gradually smaller as it approaches the posterior segments of the body. The situation of this interesting structure, relatively to that of the nervous cord, the alimentary canal, and the other organs of the body, its connexion by vascular arches with the last ventricle of the heart, and the course of the blood which it distributes, all strongly remind us of its similarity to the great aorta of vertebrata, and of the analogies which the whole of the vascular structures in these worm-like articulata bear to those of fishes and amphibia, and the earlier condition of the fœtus in the higher animals. As it passes backwards along the cord, this spinal artery gives off a pair of branches above the anterior part of each ganglion ..... until it has reached the last segment of the body, in which it is scarcely more than one third of its diameter at its origin in the third segment. Immediately above the last ganglion, the artery itself is divided into two principal branches, with only a very minute median one between them. These branches take the course of the terminal nerves of the cord, and are distributed with them to the last pair of legs and surrounding structures." (Phil. Trans. 1843, p. 283.)

Mr. Newport gives a minute description of the distribution of the lateral branches above mentioned. Each of them subdivides into four branches: of which the first three are distributed, with the three principal nervous trunks given off on each side from the ganglia, to the muscular apparatus of the body and legs; whilst the fourth, which is the smallest, proceeds exclusively, with the fourth division of the nerve, to the tracheal vessels and parts concerned in respiration. Some idea of the minuteness and extent of the arterial distribution, in animals in which no distinct blood-vessels had ever before been traced, may be formed from the fact, that Mr. Newport has ascertained the structure of the heart itself to be copiously supplied with nutrient branches; and he also states that in the peritoneum there is an extensive ramification of circulatory and tracheal vessels, intermingled with each other in the most complex manner.

In the family *Scutigeridæ,* we meet with a very interesting condition of the circulating system; which affords a still further proof of the principles advanced by Mr. Newport, touching the union of two or more original parts in the complete development of every structure. We have already seen this principle illustrated, in the progressive development of the iulidæ, two of the chambers of the heart being actually fused into one, in each moveable double segment; and we find, in the scutigeridæ, that the compound heart possesses, as its permanent structure, a condition which seems to form the obvious transition towards that of the dorsal vessel of insects.

"In this family, the number of chambers is still fifteen, as in lithobius; in which the dorsal plates of the body are alternately longer and shorter in the different segments, to the respective lengths of which the chambers of the heart are begun to be reduced. This is a condition preparatory to the union in pairs, first of the dorsal plates, and afterwards of the chambers of the heart. In the scutigeridæ, the dorsal plates are already united, and form but eight moveable coverings, one to each pair of segments, which still remain distinct on the ventral surface of the body. But although there are still sixteen chambers to the heart, the changes commenced in lithobius are carried still further in this genus; and each alternate chamber is very much smaller and shorter than the one next before it, and covered by the same dorsal plate. But although the union of the chambers has actually commenced, it is yet very imperfect, and the original divisions between them are still evident, and the systemic arteries pass off from their sides as in their imperfect state of development. But very little blood enters at the auricular orifices, which still exist in these unions. The chief part now enters through the large auricular orifices of the chambers in the middle of each dorsal plate. These are very distinct, but are placed more transversely to the heart than in scolopendra, corresponding to the more obtuse form of each chamber, and the more compact general form of the whole heart. Here then, in the gradual reduction of the number of the chambers, their compact form, and the shortening of the organ, we trace the stages of the formation of the heart in insects, in which there are seldom more than eight chambers." (Phil. Trans. 1843, p. 286.)

Mr. Newport next proceeds to investigate the Circulating apparatus of the *Scorpion,* as an example taken from one of the higher articulated classes. This animal had previously occupied the attention of anatomists; but its vascular system had been very imperfectly understood. Treviranus in 1812 described it vaguely; and first noticed the structure now described by Mr. Newport as the supra-spinal artery, as part of the nervous system, considering it a peculiarity of the nervous system of the scorpion. The same structure was afterwards noticed by Müller in 1828, but was regarded as a ligament. Both these anatomists entirely overlooked the extensive distribution of vessels from the anterior extremity of the heart in the cephalothorax. We shall not follow Mr. Newport through the minutiæ of his descriptions; but shall content ourselves with indicating some of the most important points, in which the vascular system of the scorpion differs from that of the higher myriapoda. Notwithstanding the strongly-marked diversities in external form, and in the relative development of different organs, the degree of correspondence is such, as to indicate most completely the conformity of plan in the two cases; and Mr. Newport has found the study of the comparatively simple and regular distribution of vessels in the myriapoda, an admirable key to the elucidation of the more complex and varied arrangement, which is required in the scorpion, by the more *heterogeneous* character of its general structure. Thus the great dorsal vessel, which runs as in the myriapods from one extremity to the other, only possesses the character of a heart in the anterior portion, which lies in the body of the animal; the posterior, which is contained in the tail, being contracted into a simple arterial tube. The heart is divided into eight separate chambers, which are wider in proportion to their length than in the highest of the myriapods; and which are more muscular and compact, in a degree proportionate to the greater quantity of blood to be transmitted through them, and to the force with which it is necessary to be propelled. The valves or divisions between the successive chambers are each formed by a reduplication of the whole muscular structure of the dorsal surface of the organ; but there is no complete corresponding fold, and in some chambers no fold at all, on the under surface. The reason for this imperfect structure of the valves may perhaps be found in the fact, that the blood is distributed from the heart in the scorpion in opposite directions, partly backwards to the tail, but chiefly forwards and outwards to the head and sides, as in the myriapoda; and hence it may be necessary that a reflux of blood should not be entirely prevented, as may be required in those instances in which the whole current is in one direction. The uniformity of principles on which the circulating apparatus is constructed, is remarkably displayed in the distribution of the aorta to the limbs and head, in the aggregation of segments that constitute the cephalothorax of the scorpion.

"We have seen that vessels are given off from corresponding parts in the chambers of the heart, both in the myriapoda and the scorpion; and that these vessels, the *systemic arteries*, are given to precisely similar parts in both. In the myriapoda, the anterior pair of these vessels form a vascular collar around the œsophagus in the posterior region of the head; and this also is the case in the scorpion. The median continuation of the vessel beyond this collar in the myriapoda is given to the head, to the brain, optic nerves, antennæ, and internal parts of the mouth; while the external parts of the manducatory organs, the great foot-jaws or mandibles, are supplied from the vascular collar, or from parts immediately connected with it. Now, notwithstanding the aggregation of all these parts together, as well as of the proper organs of locomotion, the trunks of the arteries still preserve their original distinctness, and enable us to identify the organs and parts of the head of the scorpion with organs that exist under other forms, although endowed with similar functions, in the more distinctly-developed head of the myriapods and insects." (Phil. Trans. 1843, p. 289.)

Thus Mr. Newport has obtained a clue to the identification of the great prehensile claws, which are the special organs of the head of the scorpion, with the foot-jaws in the centipede, and the mandibles of iulus and of insects. And in the same manner, the small prehensile organs in front may be identified as the analogues of the antennæ of insects; and these, although so remarkably altered in form, from a simple, elongated, many-jointed organ, to one of a prehensile character, still retain the same primary function—that of touching and feeling—as in their less complicated structure. The contraction of the posterior segments of the scorpion into a tail, has been already noticed as affecting the character of the posterior part of the dorsal vessel; but even the anterior portion, which serves as a heart, is also affected by it. The last two chambers, which are situated in the seventh segment and give origin to the caudal artery, are greatly reduced in size, and seem to be the means by which part of the current of blood is directed *backwards* to the tail. Each of these chambers receives its venous trunks in a direction more transversely backwards than the other chambers; so that the influx of the received blood is directed backwards. The visceral arteries from these chambers are altered in their direction; for instead of passing laterally and forwards, they give off only a small trunk forwards and to the sides of the segments, while their principal trunks are directed backwards. The distribution of the branches of the caudal artery is very similar to the course of the visceral arteries which have been described as arising from the chambered portion of the dorsal vessel,—one pair from each chamber.

The scorpion presents a system of vessels, however, specially connected with the respiratory function; of which the myriapoda seem almost or entirely destitute. The aeration of the blood, in the latter, is effected by means of *tracheæ* widely diffused through each segment; and there is no occasion, therefore, for any special vascular apparatus to bring the blood to the respiratory organs. But in the arachnida, the plan of the respiratory apparatus is different; the aerating surface being concentrated in a small number of organs termed *pulmonary branchiæ,* whose structure will be presently noticed. In order to convey the blood to these, a special system of vessels is necessary; this collects the blood from the system at large, and transmits it to the branchiæ; from which it is returned back to the heart by a large sinus. This set of vessels is termed by Mr. Newport the *portal system,* from its analogy to the portal system of vertebrata; but as its function is so different, and as it is still more analogous to the system of vessels in crustaceans and molluscs, which stand in the same manner between the systemic and respiratory capillaries, we think the use of this term objectionable, and we shall term it the *branchial* system. The centre of this system is a hollow fibrous structure, which seems to form a kind of sinus, closely surrounding the nervous cord; from the posterior part of this, the *subspinal* vessel passes off, which runs beneath the nervous cord as far as its termination; and from its sides two pairs of trunks proceed towards the first pair of respiratory organs, receiving in their course numerous small vessels from the sides of the segments. The posterior pairs of branchiæ are supplied from the subspinal vessel, by branches which in like manner collect the venous blood from the neighbouring parts; and this vessel also receives blood direct from the caudal artery or posterior prolongation of the dorsal vessel. The anatomy of the pulmono-branchiæ, (which had been previously described correctly by Müller, as being each formed of a multitude of closely-approximated thin double lamellæ, through which the blood is distributed, and brought by endosmosis into communication with the air admitted into the common cavity of the branchia, through the spiracle on the exterior of the body,) has been more minutely investigated by Mr. Newport; and his account contains some facts of interest, which we shall quote:

"Each side of these double lamellæ is formed of an exceedingly delicate and apparently structureless double membrane, which includes within it a parenchymatous tissue, formed of single vesicles or cells, in which I have been unable to detect any nuclei. These cells exhibit the appearance of simple bodies, from which it might well be conceived that vessels might be formed. In some places these vesicles are arranged more in distinct series, and are also slightly elongated. The whole parenchymatous tissue of the lamina is made up of these cells, which are larger and more elongated, and assume a slightly conical appearance near where the air enters at the base of each plate, in which part these cells are nearly uniformly distributed within the double membrane. But in the upper or more convex portion of each lamina, numbers of these double cells are aggregated together in numerous, irregular, rounded patches, which thus produce a tuberculated or glandular appearance in the laminæ. These aggregations of cells are more thickly interspersed through the structure of the lamina, the nearer they are to its convex margin, where I have sometimes seen what I believe to be delicate but exceedingly indistinct vessels penetrating the lamina, but which could be followed only for a very short distance into it, among the cells. The convex margin of each lamina is, however, bounded by a delicate but distinct vessel, which seems to form the means of intercommunication betweeen the anastomosing network of vessels distributed over the branchiæ, and the structure of the laminæ; since the delicate evanescent vessels traced into the laminæ, are derived from those which bound their convex margin. I have also observed vessels extended from these marginal vessels on the laminæ; which I regard as the anastomoses between these, and those which cover the whole branchiæ, and distribute the blood from the portal branches." (Phil. Trans. 1843, p. 296.)

The blood would seem to permeate the cellular parenchymatous tissue of each double lamella, and to be brought into relation with the air, when the branchiæ are distended during respiration, by endosmosis through the membranes, exactly as in the lungs of higher animals; the blood is then collected in sinuses, of which one originates from each lamella; and these form, by their union, a series of larger trunks or sinuses, which pass around the sides of the body in the posterior part of each segment, communicating with other vessels in their progress, and at last pouring their contents into the heart, at the auricular orifices on its dorsal surface. Besides this respiratory system of vessels, the branchiæ receive arterial branches, apparently for the nourishment of their tissues, as in higher animals. The cavities of all the branchiæ on either side (four in number) communicate directly with each other by a short narrow passage; so that the whole on one side of the abdomen forms one common cavity or lung-like organ, like the large tracheal vesicles in the abdomen of perfect insects, and thus ensures an uniformity of function at each act of inspiration.

The following is Mr. Newport's account of the course of the circulation in the scorpion; for which, we hope, our readers will now be sufficiently prepared by the anatomical details we have given:

"The blood received by the veins from the branchiæ is conveyed to the heart round the sides of the segments, receiving accessions from other segments in its course; and enters the heart at the posterior part of each chamber on its dorsal surface, through the orifices of Straus. The auriculo-ventricular cavity, dilated by the influx of blood, begins first to contract by the action of the circular fibres at the posterior part of each chamber. By this contraction, part of the blood is at once propelled laterally by the systemic arteries to the interior and sides of the body; while the remaining and chief portion is forced onwards through the valves and body of the chamber, by the successive contraction of the circular fibres, into the next chamber. A fresh accession of blood enters the heart at the auricular orifices, in the short interval of time that elapses between the contractile actions of the two chambers; which interval is probably occasioned by the reaction of the lateral muscular appendages of the organ. These contractions, commencing in the principal chamber in the sixth abdominal segment, are carried gradually onwards through the whole of the succeeding segments; so that, ere a third chamber has contracted, the first is again filled and ready to be emptied, thus occasioning by their alternate movements those pulsatory motions observed in all instances in which the heart is formed of a longitudinal series of chambers and valves, motions which are so well known in insects. The blood, propelled by these successive contractions through the dorsal vessel, is distributed to the organs in the head and thorax and the organs of locomotion. Part of it also is sent round the *aortic arches,* through the supra-spinal artery [or aorta,] *backwards* into the abdomen, giving off its minute currents for the nourishment of the cord, whilst another portion intermingled with that collected in the portal vessels is sent to the branchiæ. But its principal current still flows in the spinal artery, along the upper surface of the cord, to the terminal ganglion of the tail; where it is divided into four streams, two of which go out at the sides of the ganglion to nourish the segment, while the other two, now greatly reduced in size, proceed backwards along the terminal nerves of the cord, and becoming more and more subdivided in the last segment of the tail, are diffused through the surrounding structures. These form minute anastomoses with numerous small vessels, which gradually collecting in separate trunks on the under surface of the last segment, form the origin of the caudal portion of the subspinal vessel, which conveys the returning blood *forwards* from the tail to the abdomen, to be aerated in the branchiæ before it is again transmitted to the heart. In like manner the blood that has already circulated through the organs of locomotion, the cephalothorax and abdomen, appears to be collected in the *vena cava* which transmit it to the branchiæ before it is again employed in the circulation. Throughout the whole of its [backward] course along the [supra-spinal] artery in the tail, the blood is passed in small currents, both anterior and posterior to each ganglion, into the subspinal vessel; thus intermingling the venous and arterial blood, precisely as occurs in the abdomen. But the circulation in the caudal prolongation of the heart yet remains to be explained. We have already seen that the great dorsal artery in the tail, above the colon, forms direct vascular anastomoses around its sides, with the subspinal vessel on the ventral surface, in which the course of the blood is *forwards* to the abdomen. It is certain, therefore, that the action of the great chamber of the heart must impel the blood at once in every direction, chiefly forwards and laterally, but also in part *backwards* along the caudal artery; otherwise it would be impossible for this structure to form its anastomoses with the subspinal vein without occasioning two opposite currents in the same vessel; and this diversion of the current may perhaps be effected through the interposition of the two imperfect chambers of the heart in the last abdominal segment." (Phil. Trans. 1843, p. 298.)

Thus by his patient and minute investigations, Mr. Newport has succeeded in showing the existence of a circulating apparatus in these animals, which is scarcely inferior in extent and complexity to that of the highest vertebrata, although formed upon a very different plan. When we compare his descriptions with the vague and general terms in which the vessels of the air-breathing articulata have been until hitherto described, we cannot but acknowledge the immense advance which he has made on our acquaintance with the subject, and express our admiration of the skill and industry with which he has carried out the investigation. The entire paper, as remarked by a distinguished *Savant,** "is crowded with facts precious to science."

It now only remains for us to notice the most recently-published of Mr. Newport's Papers, "On the Reproduction of lost parts in Myriapoda

* M. Milne Edwards, in Annales des Sc. Nat.

and Insecta;" to the general results of which we must confine ourselves. It has long been known to every naturalist, that the crustacea and arachnida are capable of reproducing their limbs; and it has also been stated, that a similar reproduction of parts takes place in some of those insects which are active throughout their whole life, and do not change their form, but merely cast off their teguments and increase in size. But it has been questioned whether any reproduction of lost parts can take place in those insects, which undergo a complete metamorphosis, and which change their form, their food, and their mode of life, in passing from the young to the adult state. No investigations having been yet made, to decide the question whether the regenerative power exists in insects that undergo these changes, or in the class myriapoda, the subject has been taken up by Mr. Newport; who has arrived at the most satisfactory affirmative results. His experiments on the myriapods were of course restricted to species that inhabit our own country; and these, as is well known, are but diminutive representatives of the gigantic iuli and centipedes of warmer climates. But the evidence which they afford is quite satisfactory, especially when confirmed by appearances not unfrequently met with in preserved specimens brought from abroad. Having cut off one of the antennæ, and some of the legs, of iuli which had attained nearly their full development, he found that, at the next moult, these parts were reproduced; the new parts being much smaller and shorter, however, and the articulations of the new joints being less perfect than the old. It does not appear necessary that, for the regeneration of one part, the entire organ must be removed, as seems to be the case with the limbs of certain crustacea;* but reparation begins in the part in which the injury occurs, and the new structures produced are only sufficient to replace those which have been removed. The regenerative power seemed to be greater in those individuals, which were still adding considerably to the number of the segments at each moult, than in those which had more nearly acquired their full growth; thus in two instances there were only six joints, instead of seven, to the new antennæ; and these individuals had so nearly reached their adult state, that each acquired only one new segment to its body at this change of tegument, the succeeding change being probably the last. Similar results were obtained from experiments on the *Lithobius vulgaris*, one of the chilopoda; and in these it was seen that the reproduced legs gradually acquired the full size proper to the limbs.

"These facts sufficiently show, that a power of reproducing lost parts is quite common to the lithobii; and the frequent occurrence of legs that have not their full size, in specimens of foreign *scolopendra*, lead to the conclusion that it is equally common to these animals. There are many instances of this in the collection in the British Museum; in the majority of which, it is one of the posterior legs that has been reproduced. From the number of specimens I have met with, in which this is the case, I have been enabled to arrive at the conclusion, that although reproduced limbs in these animals may ultimately attain the full size of the normal structures, they never acquire a perfectly normal development of all their parts, which are sometimes supernumerary, but more frequently are deficient in number, and are almost invariably atrophied. This is strikingly illustrated in the development of the spines, with which the basal joint of the posterior legs of the scolopendra are armed. This fact is of consequence in a zoological point of

* See Art. I, in our present Number.

view ; as these parts have recently been much depended on in the determination of species. It is also, perhaps, of some value in relation to the laws of development; since it is found to be equally constant, as I shall presently show, in the reproduced parts of the true insecta." (Phil. Trans. 1844, p. 288.)

For his experiments on insects undergoing a complete metamorphosis, Mr. Newport selected the larvæ of the common nettle butterfly; from a considerable number of which he excised one or more of the true legs, at no very long period before they had acquired their full growth. Some of the specimens seemed to be but little affected by the operation, as they immediately began to feed very actively, even when profuse hemorrhage was going on; but on the following morning several of them had died from its results. It is interesting to remark that the hemorrhage of insects is restrained by coagulation of the circulating fluid, exactly as in higher animals; and that the rapidity with which this coagulation takes place is dependent on the comparative temperature and dryness of the atmosphere,—the hemorrhage continuing longest in air that is loaded with moisture. Beneath the eschar thus formed, the complete union of the wounded parts subsequently takes place.

"It soon became evident that even in those instances in which the insects survived the operation, their development was considerably retarded by it. This was shown by comparing the length of time that elapsed in their different states, with that required by other specimens of the same age, which had not undergone any operation before they changed to chrysalids,—the circumstances, in regard to age, quantity and quality of food, temperature, and locality in which they were placed, being in all exactly the same. In every instance the uninjured specimens prepared themselves for transformation very much earlier than those experimented on ; and they also passed much quicker into the state of pupæ. Thus, while uninjured specimens, which had not cast their last larva skin, all underwent their changes on the 29th, and morning of the 30th of May, those of the same age that had been first subjected to experiment did not undergo a corresponding change of skin until late on the 31st. Precisely the same thing occurred with the more matured larvæ, all of which were retarded in their change to the pupa state beyond the time at which others of the same age had entered it." (Phil. Trans. 1844, p. 290.)

Some of the larvæ, when experimented on, had already entered their last skin; and in these there was no opportunity of observing the progress of the reparative operation until the metamorphosis into the pupa state. Most of those which then remained alive gave distinct evidence that the process of regeneration was taking place; the tibial and tarsal portions of the legs having been restored in some, and the entire limbs in others; while in two or three instances the experiment appeared to have entirely failed. In the second set of larvæ, which had to undergo another moult before passing into the pupa state, indications were seen that the reparative process had commenced even then; a slight enlargement being seen at the divided surface, and, in one instance, the leg which had been removed at the tibial joint being reproduced with the tarsus and claw. In butterflies produced from both sets of larvæ, the reproduction was complete in some cases, and less complete in others; whilst in some specimens, produced from larvæ of both ages, there seemed to be no attempt at reproduction. In one of the specimens, from which two legs had been removed, the second leg was reproduced, whilst the third was not. This fact, taken in connexion with the entire absence of reproduction in certain other cases, might appear to indicate that the reproduction of lost parts depends on the existence of special structures, situated in some portion of the base of the limb (as in crustacea); the removal of which prevents the redevelopment of the lost part. But such a conclusion would be inconsistent with other results of the same series of experiments; for even when the entire limb, with the coxa or basilar joint, was removed, a new limb was produced in some cases, the merest possible rudiment of a limb developed in others, whilst in others there was no indication of it whatever.

"When an entire limb is reproduced, it is always composed of its *essential* parts,—coxa, femur, tibia, tarsus, and claw. Besides being inferior in size, the tarsus is often deficient in the number of its joints; and the *subsidiary* parts of the limb, the articular spines, are almost always absent. This is more especially the case when a limb has been divided in the femoral or tibial joints; and the reproduction of the limb has, in consequence, been only partial. But in all these cases, the terminal part of the organ, the *claw*, is invariably reproduced. This was strikingly shown in some specimens of partial reproduction, in which the claw was formed at the extremity of a metatarsal joint, or when one or more of the tarsal joints were wanting." (Phil. Trans. 1844, p. 292.)

There is no reason to believe that the reproduction of lost parts ever takes place in myriapoda which have undergone their full development, or in insects which have passed through their metamorphoses. But we think that the negative requires to be proved; and we recommend the subject to Mr. Newport's attention. All the knowledge we at present possess on this subject would lead us to believe that the reproductive process, in the two classes just named, is confined to the time when the nutritive operations are actively going on, either for the development of new parts, or for the completion of the structures already in progress. There is reason to think that, in insects, the wings are not even supplied with blood, for any long period after the final change; so that a reparation would be there impossible. To what extent it may be carried in other parts would form an interesting subject of inquiry.

But we must now close our notice of these interesting Researches. We have entered more fully into the consideration of them, firstly, because of the very high value we attach to inquiries of this kind, when pursued with a philosophical spirit, as leading us to a knowledge of facts and principles of the greatest possible importance in connexion with those laws of life on which alone all sound practice of medicine and surgery is established; and next, because these researches of Mr. Newport have not hitherto received that attention, in this country, which we are confident they merit. This neglect may, in part, be attributed to the minuteness of the subjects of the inquiry, and also to the very little *practical* knowledge of the natural history of these subjects possessed by even our most distinguished medical men, who alone are fitted by education, in all other respects, for prosecuting and fully appreciating the value of such inquiries. It is the very minuteness of the subjects, however, which, while it increases a thousandfold the difficulties of investigating them, gives them,—especially in combination with their natural history,—much of their philosophical value. It is only in the minute forms of creation in which the analogues of the complicated structures of our own bodies exist in their simplest condition, that we are enabled to learn, as it were, the alphabet of organized life; and where, by tracing the changes which these simple conditions undergo, in proceeding from the lowest to the highest forms, we are enabled with certainty to combine the hieroglyphical facts they afford into principles and laws demonstrative of one uniform and magnificent plan of creation; and thus ultimately come to read the most intricate problems of health and disease, which are the great object of all medical science.

We refer with pleasure to the Researches before us for some illustrations of our position:—to the elucidation we have now, for the first time, afforded to us, in Mr. Newport's investigations, of the mode in which the brain itself is formed in the early condition of the embryo in the articulata (and by inference also in the vertebrata) by the coalescence of the ganglia, or nervous centres, of a definite number of rudimentary segments that compose the head; to the demonstration of the plan on which the circulatory system is formed in the same class, and its conformity with that of vertebrata, in the existence of aortic arches, cephalic arteries, and descending aorta, as applicable to the abnormal surgical anatomy of the arteries in the vertebrata; and, lastly, to the facts connected with the reproduction of lost parts, which in like manner are applicable in principle to the healing of wounds and reparation of structures. We might mention many other good results that may be derived from minute anatomical investigations, combined with a practical knowledge of the natural history and habits of the objects themselves, by those who are otherwise fitted for such investigations. We trust that, in future, more attention will be devoted, in our medical schools, to those important branches of science—the anatomy and habits of the inferior animals; and that watching the vagaries of the summer fly, or feeding and rearing the caterpillar, or studying the habits of "the poor beetle that we tread upon," may cease to be regarded as totally useless, or as too contemptible for a philosopher, because not *immediately* applicable to some practicable purpose.

---

### Art. XX.

*Transactions of the Provincial Medical and Surgical Association.* Vol. XIII.—*London*, 1845. 8vo, pp. 418.

